# Overview of Huntington’s Disease and Emerging Treatment Strategies: A Narrative Review

**DOI:** 10.7759/cureus.98243

**Published:** 2025-12-01

**Authors:** Alexis J Vega, Gabriel V Hernandez, Pearse A O'Malley, Connor J Robin, Amanda N Parra, Giustino Varrassi, Sahar Shekoohi, Alan D Kaye

**Affiliations:** 1 Neurology, Tulane University School of Medicine, New Orleans, USA; 2 Internal Medicine, Tulane University School of Medicine, New Orleans, USA; 3 Medicine, Tulane University School of Medicine, New Orleans, USA; 4 Medicine, Louisiana State University Health Sciences Center, New Orleans, USA; 5 Medicine, Ross University, Miramar, USA; 6 Pain Medicine, Fondazione Paolo Procacci, Rome, ITA; 7 Anesthesiology, Louisiana State University Health Sciences Center, Shreveport, USA

**Keywords:** aso, autosomal dominant, crispr, ferroptosis, huntington’s disease, sirna, sirtuins, striatum

## Abstract

Huntington’s disease (HD) is an autosomal dominant, progressive neurodegenerative disorder caused by a cytosine-adenine-guanine trinucleotide repeat expansion in the huntingtin (HTT) gene. The symptoms of HD include severe motor dysfunction, cognitive issues, and emotional dysregulation. These combined issues are not only debilitating but also lead to depression/anxiety, increased suicide rates, and caregiver burnout. Our narrative review summarizes several recent studies examining the efficacy and differences among emerging treatment strategies for HD. A systematic search of peer-reviewed literature was conducted, focusing on recent studies that describe molecular genetic manipulation of the HTT gene/huntingtin protein. The results of our narrative review reveal potential benefits in slowing disease progression and enhancing symptomatic management through genetic silencing, gene editing using Clustered Regularly Interspaced Short Palindromic Repeats technology, antisense oligonucleotide-mediated protein suppression, RNA interference, sirtuin modulation, and ferroptosis inhibition. Future studies should aim to examine the disease progression of HD models using multimodal therapeutic options with efficient delivery methods to deep brain structures, as well as to develop biomarkers to track disease progression and treatment response.

## Introduction and background

Huntington’s disease (HD) is a progressive neurodegenerative disorder caused by a mutation in the HTT gene, leading to the production of a non-functional huntingtin protein. This protein causes brain degeneration, particularly in areas responsible for motor control, cognition, and emotional regulation. Symptoms typically appear between the ages of 30 and 50, although juvenile forms can develop earlier [1]. As the disease progresses, patients experience motor dysfunction, cognitive decline, and significant personality changes, including irritability and social withdrawal.

Mental health issues, particularly depression, are common in HD. The emotional distress caused by the disease's progression can lead to feelings of hopelessness and frustration. Depression in HD can also be linked to the neurobiological changes caused by the disease, with some evidence suggesting that the areas of the brain affected by HD are also involved in mood regulation. Anxiety, irritability, and mood swings are also prevalent, further exacerbating the emotional toll.

HD patients are at an elevated risk of suicide in comparison with the general population [2]. The genetic nature of the disease compounds this elevated risk, as individuals often face the agonizing decision of whether to undergo genetic testing, knowing that a positive result could mean they will inevitably develop the disease. This knowledge, coupled with the progressive nature of HD, creates a constant psychological burden on both patients and their families, leading to heightened levels of stress, anxiety, and depressive symptoms. The impact of HD extends beyond the patient to their family members. As HD is inherited in an autosomal dominant pattern, family members often live with the uncertainty of whether they will inherit the gene, which can lead to emotional and psychological stress for future generations. The caregiving burden is also substantial, as families are tasked with providing increasing levels of care as the disease progresses [3].

Currently, there is no cure for HD, and treatment focuses on managing symptoms. However, recent research into the genetic mechanisms of HD has led to promising studies on epigenetic therapies that may slow or reverse disease progression [4]. These therapies aim to regulate the expression of the mutated gene, potentially reducing the production of the toxic huntingtin protein and mitigating its effects.

Effective treatments for HD would not only improve physical symptoms but could also reduce psychiatric symptoms, such as depression and anxiety, thereby lowering the risk of suicide. For families, effective treatments would alleviate the emotional distress and uncertainty associated with caring for a loved one with HD, offering hope for future generations. Emerging therapies, such as epigenetic treatments, could particularly help manage mental health symptoms by targeting the biological causes of depression and anxiety, leading to improved cognitive and emotional functioning and a better quality of life for both the patients and caregivers. This review article compares several recent studies to examine the efficacy and differences among emerging treatment strategies for HD. A systematic search of peer-reviewed literature was conducted, focusing on recent studies that describe molecular genetic manipulation of the HTT gene/huntingtin protein. Understanding the molecular genetic mechanisms of HD can help develop novel treatment strategies to slow disease progression and ultimately improve patients' quality of life.

## Review

Epidemiology, genetics, and pathophysiology

In a meta-analysis of 33 studies published from 2010 to 2022, the worldwide incidence and prevalence of HD were estimated at 0.48 cases per 100,000 person-years and 4.88 per 100,000 persons, respectively [5]. The studies under review pooled data from 21 countries to form subgroup analyses by continent [5]. In all subgroup analyses, the Asian and African subgroups reported significantly lower incidences and prevalences of HD than studies conducted in Europe, North America, and Australia [5,6]. Prevalence and incidence were not constant across European countries, with some exhibiting lower and others higher prevalence of disease than neighboring countries. According to studies measuring HD prevalence in these countries, the discrepancies between neighboring countries may be due to a local founder effect, leading to populations with extremely high or low CAG trinucleotide tract sizes.

In addition to the founder effect, differences in the prevalence and incidence of HD may also be attributed to discrepancies in health care, including the availability and affordability of molecular testing [5,7]. Furthermore, populations with higher prevalence and incidence of HD than their counterparts can be explained by differences in their huntingtin gene haplotypes [5,6].

Genetics

The huntingtin (HTT) gene is located on chromosome 4p16.3, which consists of unstable CAG repeats in the first exon that encode a polyglutamine tract near the amino terminus [5,7,8]. This gene encodes huntingtin, a large protein that exhibits polymorphic variation in the general population. The HTT gene is inherited in an autosomal dominant manner and plays a critical role in anticipation of HD [8]. Anticipation is a phenomenon in which individuals with a disorder often display earlier clinical onset of symptoms in successive generations. This phenomenon is exacerbated in most autosomal dominant genetic disorders [7,8].

No cases of HD have been reported in individuals with 26 or fewer trinucleotide repeats in their HTT gene; thus, they are in the normal range [5,7]. The normal, mutable range of CAG repeats indicates that tracts within this range could expand during meiosis, potentially passing on the HD phenotype to offspring [5,7]. In individuals with 36-39 CAG repeats, incomplete penetrance of HD is reported, but complete penetrance is found when one possesses 40+ CAG repeats [5,6,7]. While the reason is unclear, the most common normal repeat number is 17, while the most common abnormal number is 43 [7]. HD has a strong inverse relationship between repeat length and the age of disease onset [5,8]. Expansion of the HTT trinucleotide tract usually is by only one to a few repeats per generation but can still explain anticipation in successive generations [8]. Most children with HD have been found to have CAG repeat numbers of 50+ [7].

Pathophysiology

Although the pathophysiology of HD is not yet fully understood, it is known that a gain of toxic function because of the expanded polyglutamine tract is responsible [9]. The ubiquitous expression of the HTT makes it challenging to determine its specific function; however, it is acknowledged to be valuable in multiple cellular processes, including protein clearance, protein-protein interactions, mitochondrial function, axonal trafficking, gene transcription, and post-translational modifications [9,10]. In animal studies reviewed by Li and Li, mutant HTT (mHTT) proteins (those with abnormally high glutamine residues) formed aggregates in the nuclei and cytoplasm of brain cells, leading to abnormal interactions with other proteins [9,10]. HTT aggregates are also hypothesized to impair endocytosis physically and vesicular trafficking in neuronal processes and axon terminals [9]. Oxidative phosphorylation enzymes of the inner mitochondrial membrane exhibit decreased activity in patients with HD, suggesting a role in mitochondrial dysfunction in brain cells [10]. Finally, abnormal post-translational modifications caused by HD affect ubiquitination, phosphorylation, sumoylation, palmitoylation, and acetylation of cellular proteins, leading to further accumulation of htt protein in brain cells [10].

Clinical manifestations and current management

Currently, there is no cure for HD, and treatment primarily focuses on symptomatic improvement. According to the American Academy of Neurology guidelines, pharmacological interventions mainly target hyperkinetic movement symptoms such as chorea and dystonia, as well as associated psychiatric and cognitive disturbances [11]. For chorea, tetrabenazine is considered a first-line therapy [12,13]. It acts by reversibly inhibiting the vesicular monoamine transporter 2 (VMAT2), thereby selectively depleting dopamine in the central nervous system (CNS). While effective, tetrabenazine carries potential side effects, including depression, fatigue, akathisia, insomnia, and somnolence. These adverse effects are most commonly seen during treatment initiation. Given that HD patients are already at higher risk for depression and suicidal ideation, close psychiatric monitoring is essential when prescribing this medication [13]. Other dopamine antagonists, such as olanzapine, quetiapine, and aripiprazole, have also been used with success in treating both motor symptoms and psychiatric manifestations. However, aripiprazole and similar agents may lead to side effects like akathisia and tardive dyskinesia. Amantadine, an NMDA receptor antagonist, has shown some efficacy in reducing chorea at higher doses. Riluzole, a glutamate release inhibitor, is currently under investigation for its potential neuroprotective role. However, conclusive clinical benefits have not yet been established.

Parkinsonian features in HD patients may respond to conventional dopaminergic therapies such as levodopa, dopamine agonists, and amantadine. Additionally, botulinum toxin injections are effective in managing focal dystonias. Psychiatric symptoms, particularly depression, are common and should primarily be managed with non-pharmacological interventions, including counseling and psychosocial support, as evidence for the benefit of selective serotonin reuptake inhibitors and tricyclic antidepressants in HD-related depression remains limited. Patients are advised to avoid tobacco and alcohol, which can exacerbate symptoms. Supportive care plays a vital role and includes attention to nutritional needs, physical therapy, nursing support, and adaptive equipment to help maintain patient safety and independence [11].

Emerging treatment strategies

Significant advancements in research hold promise for novel treatment strategies for HD. One emerging area of research is gene therapies. A mutation in the HTT gene causes HD, leading to the production of a harmful protein that damages neurons [14,15]. Gene therapy aims to target and reduce the expression of the mHTT gene. One approach involves using allele-specific small interfering RNA (siRNA) to silence the mHTT gene [14-17]. A subsequent study confirmed the initial findings by showing that a single striatal siRNA targeting the mHTT gene effectively silenced it. This leads to reduced behavioral and neurological irregularities in a rapid-onset viral transgenic HD mouse model.

Additional studies using fibroblasts derived from human HD patients have demonstrated that allele-specific siRNA can selectively silence the mHTT allele while preserving the normal allele. This approach has shown effective reduction of mHTT expression in vitro, and these findings support the potential clinical applicability of allele-specific RNA interference (RNAi), particularly in patients with heterozygous single-nucleotide polymorphisms (SNPs) suitable for selective targeting [16,17]. Population-based studies estimate that up to 75% of HD patients carry SNPs that may make them eligible for this individualized therapy, highlighting its real-world clinical relevance as a disease-modifying approach [16].

Non-allele-specific CRISPR/Cas9 gene editing has also been investigated as a potential strategy for eliminating mHTT expression. The CRISPR/Cas9 system, derived initially from bacterial immune defense mechanisms, uses a guide RNA and a Cas9 endonuclease to target specific DNA sequences, including those encoding expanded CAG repeats [14,15,18,19]. Preclinical studies have successfully used CRISPR to excise or disrupt the polyglutamine-encoding region of mHTT in both cellular and mouse models, demonstrating reductions in toxic protein aggregates and reactive astrocytosis [15,20-22]. However, translation to human clinical studies remains limited. A significant concern is the potential off-target effects and the unintended loss of wild-type HTT, which plays essential roles in adult neuronal function and systemic homeostasis [19,21-23]. At present, CRISPR-based strategies for HD remain in early preclinical stages, and no human trials have yet been conducted. Nonetheless, the approach holds promise for future development as delivery methods and allele specificity improve [23].

Gene-silencing therapies targeting mHTT, including antisense oligonucleotides (ASOs), adeno-associated virus (AAV)-mediated RNAi, and emerging gene-editing technologies, represent promising strategies for HD treatment [14,16,19]. ASOs are short single-stranded oligomers composed of chemically modified nucleotides that bind to RNA to modulate its function and have demonstrated effective suppression of HTT expression in the CNS of HD patients [15,19]. A notable study by Tabrizi et al. showed that ASO-mediated reduction of huntingtin protein was well tolerated in patients with neurodegenerative disease [15]. In parallel, AAV-mediated RNAi therapies have yielded favorable results in animal models, with reductions of huntingtin levels by up to 60%, accompanied by improvements in motor function and neuropathology, without significant toxic effects [19-21]. The AMT-130 trial, which employs AAV5-miHTT to deliver RNAi, is currently ongoing to assess safety and efficacy in early-stage HD patients, marking a significant step toward clinical translation [19]. Other AAV-based therapies remain in preclinical or early clinical stages, showing promise for targeted mHTT reduction but requiring further validation in human trials [14,16]. It is important to note that huntingtin is essential for normal development, as its complete knockout in mice results in severe outcomes, including acute pancreatitis [15,16,22,24], underscoring the need for allele-specific or partial suppression approaches. Overall, advances in gene therapy, spanning ASOs, AAV-mediated RNAi, CRISPR technology, and prime editing, offer hope for individualized, disease-modifying treatments in HD by efficiently targeting and degrading the mHTT protein [14,16,19].

Another epigenetic treatment approach for HD involves sirtuins. These proteins, influenced by the NAD+/NADH ratio, have emerged as potential players in HD [14,25,26]. Sirtuins participate in autophagy by inhibiting protein acetylation and demonstrate neuroprotective characteristics, primarily mediated by SIRT1 [25,26]. SIRT1 regulates neurotrophic factors, such as brain-derived neurotrophic factor (BDNF), and suppresses apoptosis, thereby contributing to its neuroprotective effects [14]. The cerebral cortex and hippocampus receive support from BDNF through modulation of synaptic plasticity, promotion of neuronal survival, and influence on cellular differentiation [14]. SIRT1 also reduces the mitochondrial damage induced by the mHTT protein by deacetylation of peroxisome proliferator-activated receptor gamma coactivator 1-α (PGC-1α) [25]. A study by Lee et al. and other supporting preclinical studies indicate that increasing sirtuin levels could be advantageous in HD models, as shown by improved survival rates and reduced striatal atrophy [14,25,26]. Notably, the effects of sirtuins may vary by gender, underscoring the importance of further research to understand their therapeutic implications fully [14].

Dhingra and Gaidhane further characterize the epigenetic innovations developed to manage HD symptoms and disease progression. This review examined studies on the pathophysiology, phenotypic presentation, and available therapeutics for HD, again highlighting the emphasis on symptom reduction that current treatments provide to patients [27]. Prior investigations into the use of cholinesterase inhibitors, such as donepezil and rivastigmine, have had limited utility in reducing choreiform motor symptoms of HD. Still, ongoing studies are assessing the efficacy of agents other than tetrabenazine. One potential approach uses zinc finger proteins (ZFPs), engineered DNA-binding proteins that can bind to the HTT promoter region to induce transcription and modulate cellular activity. However, there is concern about inadvertent gene expression arising from ZFP binding to alternative gene promoters. Induced pluripotent stem cell (iPSC) treatment, which involves reprogramming an individual's stem cells to differentiate into a desired cell type, such as neurons, may also be helpful; however, mutations within the iPSCs may develop, risking tumor formation [27]. Furthermore, in a study in which mouse fibroblasts were induced into neural stem cells before autologous transplantation, the mice showed no significant improvement in motor function despite iPSC-derived neuronal differentiation [28]. Given the debilitating chorea that may arise from HD, there is an urgency to find practical therapies that may address these motor symptoms.

The authors note that benzodiazepines and flavonoid compounds may offer short-term relief of motor symptoms in acute settings, but efforts must be made to identify longer-term management options. Monoclonal antibody therapy has been proposed to reduce symptoms by reducing huntingtin protein expression by introducing a mutant form into cells via a viral vector. Pridopidine is an experimental drug that acts as a sigma-1 receptor agonist and may reduce neuronal apoptosis in individuals with HD; it may also protect against mHTT-induced apoptosis and therefore be used in conjunction with mAB therapy. Lastly, DNA repair mechanisms, such as nuclease expression encoded by the FAN1 gene, are thought to be targets for therapy, as increased FAN1 and nuclease expression can reduce CAG repeats and, subsequently, HD symptom progression [28]. Nevertheless, these investigations are nascent, and their practical use in a clinical context remains uncertain.

While the pathophysiological mechanisms of HD-induced neurodegeneration are not entirely understood, there is evidence to suggest that aberrant neuronal cell death plays a primary role in disease progression. In this regard, Costa et al. note that ferroptosis, characterized by increased intracellular iron and reactive oxygen species (ROS) that trigger apoptosis via lipid peroxidation, is suspected to be involved in HD pathogenesis [29]. It is well established that mHTT increases abnormal protein accumulation and lipid peroxidation in striatal neurons, leading to subsequent apoptosis. Additionally, plasma iron appears to accumulate in the neuronal mitochondria of HD mouse models [30]. Additionally, glutathione is an antioxidant that reduces intracellular ROS, limiting damage to cell membrane lipids; it has previously been shown that individuals with HD have significantly reduced plasma glutathione levels. Together, these results suggest that neuronal cell death in HD is accelerated by ferroptosis and is likely driven by the mHTT gene. There is interest in investigating ferroptosis inhibition as a therapeutic approach for patients with HD, as reduced intracellular iron and ROS accumulation may be neuroprotective [29,30]. Upstream inhibition includes reducing plasma iron availability, which can be achieved with iron chelators such as deferoxamine. However, it is unclear whether treatment with iron chelators reduces the rate of neurodegeneration, as no clinical studies have addressed this hypothesis. Ferroportin, a cell membrane protein that transports iron to the extracellular space, is another target of study, as its downregulation is implicated in the pathogenesis of other neurodegenerative disorders, e.g., Alzheimer’s and Parkinson’s diseases. Nevertheless, further evidence of clinical improvement in mouse models following modified iron homeostasis is required to elucidate appropriate pharmacotherapies for HD (Table 1) [30].

**Table 1:** Recent studies examining potential novel treatment strategies for HD HD: Huntington’s disease, ASO: antisense oligonucleotide, CRISPR: Clustered Regularly Interspaced Short Palindromic Repeats

Study	Groups/models	Results/findings	Conclusions
Kim et al. (2021) [14]	Humans (clinical trials), HD animal (mice), and cell models	Gene silencing, neurotropic support, and stem cells show promise	Early, combined treatment with improved delivery and specificity is warranted
Fields et al. (2021) [16]	Allele-specific and non-specific DNA/RNA targeting	Silencing, prime editing allows precise DNA correction	Gene targeting, allele-specific, and prime editing show promise
Tabrizi et al. (2019) [15]	Human and animal models	mHTT disrupts transcription, CSF mHTT is lowered by ASO-mediated protein suppression	ASO-mediated protein suppression shows promise. Biomarkers needed for early tracking of disease
Tabrizi et al. (2022) [19]	Humans, preclinical models	ASO trial termination should warrant further learning, HTT targeting, non-allele vs allele selectivity, nucleic acid therapeutics, gene therapy approaches, AAV-mediated RNAi therapy, splicing modulators, and DNA repair	Most promising approaches are ASOs, gene therapy, and small molecules, which need effective delivery to the brain and risk/benefit analysis
Bashir (2019) [21]	clinical/preclinical ASOs, RNAi, ZFPs, CRISPR; immunotherapy stem cells	ASOs in manifest trials, RNAi, ZFPs, and CRISPR preclinical	Proximal HTT targeting is key, allele specificity safety unknown; delivery/biomarkers needed
Lee (2019) [25]	Mammalian neurodegeneration models	Sirtuins deacetylate autophagy proteins; overexpression aids HD models	Activating sirtuin-regulated autophagy is neuroprotective
Dhingra (2023) [27]	HD patients, preclinical models	Review of novel HD drugs and treatments	Gene therapy and ferroptosis inhibitors show promise
Costa (2023) [29]	Brain diseases with ferroptosis	Explained ferroptosis mechanisms in neurodegeneration	Targeting ferroptosis may treat brain disease
Mi (2019) [30]	HD models	Ferroptosis drives HD neurodegeneration	Ferroptosis inhibition is a potential HD therapy

Discussion

HD is an autosomal dominant neurodegenerative disorder characterized by progressive motor, cognitive, and psychiatric impairments, leading to significant emotional distress, increased suicide risk, and caregiver burden [2,4]. Currently, only symptomatic treatments exist, such as tetrabenazine for chorea and antidepressants for psychiatric symptoms, but these do not alter disease progression [4,11]. Recent advances in molecular biology have shifted the therapeutic focus toward disease-modifying approaches targeting the mHTT gene and its downstream effects.

Our narrative review highlights several promising therapeutic strategies centered on molecular genetic manipulation of HTT. Gene silencing approaches, including ASOs, have shown efficacy in lowering mHTT protein levels in the CNS of HD patients, as demonstrated by Tabrizi et al. in a landmark 2019 clinical trial, in which ASO administration resulted in a dose-dependent reduction of mHTT in cerebrospinal fluid, with a favorable safety profile [15,19]. Similarly, AAV-mediated RNAi strategies, such as the ongoing AMT-130 trial, employ viral vectors to deliver microRNA targeting HTT mRNA, achieving up to a 60% reduction in huntingtin levels in preclinical models and early-phase human studies, with improvements in motor function and neuropathology reported in animal models [19-21]. These approaches, however, face challenges, including delivery to the deep brain structures and avoidance of off-target effects.

In addition to gene silencing, CRISPR-Cas9 gene editing has been explored to selectively excise the expanded CAG repeat region in the HTT gene. Preclinical studies in HD mouse models demonstrated that CRISPR-mediated deletion of the polyglutamine (polyQ) domain reduced mHTT aggregates and reactive astrocytosis, suggesting a potential for long-term disease modification [14,16,20]. However, variability in editing efficiency and concerns about collateral damage to the wild-type HTT gene, which is essential for neuronal survival and development, highlight the need for allele-specific or carefully controlled editing protocols [15,16,22,24].

Stem cell therapies represent another promising avenue. Studies using iPSCs derived from HD patients have shown potential to replace lost neurons and restore function, with early animal experiments reporting improved motor coordination and reduced striatal atrophy [26]. However, clinical translation is limited by challenges in cell differentiation, integration, and long-term survival.

Neuroprotective strategies focusing on apoptosis inhibition and enhancing cellular resilience have gained attention, especially those targeting sirtuins (SIRT1), which regulate cellular metabolism and stress responses. Jeong et al. demonstrated that SIRT1 activation confers neuroprotection by modulating transcriptional pathways, suggesting a role for sirtuin-activating compounds as adjunct therapies [26]. Additionally, recent research into ferroptosis, an iron-dependent form of programmed cell death implicated in neurodegeneration, suggests that iron chelators might mitigate oxidative damage in HD brains, with preclinical studies reporting reduced neuronal death and improved behavioral outcomes following treatment [30].

HD is an ideal candidate for genetic therapies due to its monogenic etiology involving the HTT gene, simplifying therapeutic targeting compared to other movement disorders with complex genetic backgrounds [31]. Nonetheless, most of the studies reviewed remain at early preclinical or phase 1/2 trial stages, with limited data on long-term efficacy or clinical implementation. Furthermore, combination therapies that integrate genetic, epigenetic, and neuroprotective approaches may be necessary to address HD’s multifactorial pathophysiology [14] in its entirety.

Future research should prioritize refining delivery methods for gene and epigenetic therapies, especially targeting the striatum, where HD pathology is most severe. Improvements in viral vectors or novel delivery systems could enhance efficacy and durability, as observed in ongoing AAV-RNAi clinical trials [14,19,21]. Finally, development and validation of reliable biomarkers, such as cerebrospinal fluid huntingtin levels, will be crucial for early diagnosis, monitoring therapeutic response, and optimizing treatment regimens [15,19,21].

## Conclusions

The disabling motor, psychiatric, and systemic manifestations of HD lead to a diminished quality of life for patients and their families. Physicians should consider the complexity of the disease when caring for this patient population. Doctors should take an individualized approach that addresses not only the patient’s motor symptoms but also their behavioral comorbidities, and families should be included in decisions when possible. A comprehensive understanding of HD's intricate pathology is crucial for developing a therapy that can address its multifaceted nature, making continued investigation and collaboration in this field imperative. Ongoing trials of gene therapy targeting the HTT gene and epigenetic therapies for neural protection are crucial, as they have the potential to improve patients' quality of life.

Additionally, the ability to slow disease progression or cure HD would likely prevent emotional turmoil and suicide rates for future generations. This is because gene therapies can make HD more manageable, leading to less hopelessness among patients and less neuronal destruction. The perseverance of researchers in their quest to find a cure for HD is inspiring, and their groundbreaking discoveries bring us closer to a future where effective treatments can improve the lives of affected patients, their families, and future generations.
